# Crossing the Barrier: A Comparative Study of *Listeria monocytogenes* and *Treponema pallidum* in Placental Invasion

**DOI:** 10.3390/cells13010088

**Published:** 2023-12-31

**Authors:** Samuel J. Eallonardo, Nancy E. Freitag

**Affiliations:** 1 Department of Microbiology and Immunology, University of Illinois Chicago, Chicago, IL 60612, USA; seallo2@uic.edu; 2 Department of Pharmaceutical Sciences, University of Illinois Chicago, Chicago, IL 60612, USA

**Keywords:** vertical transmission, pregnancy, *Listeria monocytogenes*, *Treponema pallidum*, syphilis, congenital syphilis

## Abstract

Vertically transmitted infections are a significant cause of fetal morbidity and mortality during pregnancy and pose substantial risks to fetal development. These infections are primarily transmitted to the fetus through two routes: (1) direct invasion and crossing the placenta which separates maternal and fetal circulation, or (2) ascending the maternal genitourinary tact and entering the uterus. Only two bacterial species are commonly found to cross the placenta and infect the fetus: *Listeria monocytogenes* and *Treponema pallidum* subsp. *pallidum*. *L. monocytogenes* is a Gram-positive, foodborne pathogen found in soil that acutely infects a wide variety of mammalian species. *T. pallidum* is a sexually transmitted spirochete that causes a chronic infection exclusively in humans. We briefly review the pathogenesis of these two very distinct bacteria that have managed to overcome the placental barrier and the role placental immunity plays in resisting infection. Both organisms share characteristics which contribute to their transplacental transmission. These include the ability to disseminate broadly within the host, evade immune phagocytosis, and the need for a strong T cell response for their elimination.

## 1. Introduction

In principle, a fetus is a promising target for a bacterial pathogen. The immature fetal immune system is still developing and is ill prepared to combat infection. The maternal immune function is altered so as to prevent a defense response against fetal alloantigens. The fetus and placenta are highly perfused and provide a nutrient-rich environment. However, invading bacteria must first manage to reach the fetus to take advantage of this niche, and that necessitates the ability to cross the maternofetal barrier.

The term vertical transmission refers generally to any transmission of infection from mother to fetus prior to birth. There are two main routes by which an infection can be transmitted to a fetus during pregnancy: the pathogen can directly cross the placental barrier or it can enter the uterus by ascending the maternal genitourinary (GU) tract. As might be expected, GU-colonizing pathogens, such as *Neisseria gonorrhea*, *Chlamydia trachomatis*, and *Streptococcus agalactiae*, are commonly transmitted from mother to child via the GU route [1,2]. In contrast, the crossing of the placental barrier requires either bloodstream transmission and/or pathogen invasion of placental cells. This review will focus on the transplacental transmission of bacteria, a facet of infection that is restricted to a very small number of bacterial pathogens.

Relative to the number of viruses and parasites known to be transmitted transplacentally, the ability to directly invade and cross the placenta is quite rare among bacteria. Of all the bacteria capable of causing human disease, only two are commonly transmitted transplacentally: *Treponema pallidum* subspecies *pallidum* and *Listeria monocytogenes*. This observation is notable because the list is so short and as such serves to emphasize the effectiveness of the placenta barrier in keeping maternal and fetal compartments separate. It also raises the question as to why two bacteria that do not appear to be particularly similar in many respects are able to defeat placental defenses. *T. pallidum* is a spirochete that causes chronic sexually transmitted infections [3] and *L. monocytogenes* is a Gram-positive acute gut pathogen [4]. This article provides a focused literature review of how these two bacterial pathogens navigate the maternal–fetal barrier and how the maternal immune response influences the course of disease. Through the comparison of these two organisms, insights arise regarding functional mechanisms that contribute to enable bacterial access to the placenta and fetus.

## 2. Immunity at the Maternal–Fetal Interface

The maternal–fetal interface in the placenta represents a unique immune environment not otherwise present during a typical bacterial infection. At all times, the immune response must navigate the twin dangers of failure to control pathogen replication and autoimmune responses against fetal antigens. The primary immune cells present are decidual natural killer (NK) cells (dNK), CD8+ T cells, and Hofbauer cells, which are placental macrophages [5].

Decidual NK cells are a specialized population of NK cells present in the decidua, the part of the uterus in contact with the placenta [6]. They are distinct from circulating NK cells and play important roles in implantation and vascular remodeling necessary for a healthy pregnancy. dNK cells produce cytotoxic granules but demonstrate low cytotoxicity compared to peripheral NKs and are not efficient at killing MHC-1 deficient cells [7,8]. This may be due to interactions with HLA-G-producing extravillous trophoblasts, placental cells that invade the decidua [9]. dNKs are known to secrete high levels of granulysin, an antimicrobial peptide, and may play a direct role in modulating antiviral immunity [6,10]. The role of dNKs in antibacterial immunity is less understood.

CD8+ T cells are present in the healthy decidua representing 2–7% of all CD45+ cells, primarily belonging to the effector memory subtype [11,12]. These cells exhibit a complex phenotype, mixing both dysfunction and activation. Notably, they highly express inhibitory checkpoint molecules PD1 and CTLA4. They also have low expression of effector molecules perforin and granzyme B but high granulysin [12]. However, when stimulated ex vivo, the cells are still capable of cytotoxicity and producing cytokines such as IFN-γ [12,13].

Hofbauer cells are resident placental macrophages that are present in the placenta for almost the entirety of gestation [14]. Unlike CD8+ T cells and dNKs, they are derived from the fetus and are located in the placenta itself rather than the decidua [15]. They are typically localized between the trophoblast layer and fetal blood vessels [16]. These cells are important in regulating the development and morphology of the placenta, especially the blood vessels [17,18,19]. They exhibit a complex phenotype but are primarily characterized as M2 macrophages. They are not thought to be directly microbicidal but can influence the immune response through cytokine production [20].

## 3. *Treponema pallidum* subsp. *pallidum*

### 3.1. Epidemiology

*Treponema pallidum*, subspecies *pallidum*, is the causative agent of syphilis and has been known to infect the fetus for at least 500 years, causing a clinical syndrome known as congenital syphilis [21]. *T. pallidum* is an obligate human pathogen and is spread by sexual intercourse or similarly close contact. Without treatment, the bacteria can remain resident in the body indefinitely. A woman who becomes pregnant while infected with *T. pallidum* is at risk of transmitting the disease to the fetus, resulting in congenital syphilis. Thanks to the availability of antibiotic treatment and public health efforts, US rates of congenital syphilis were as low as 8.4 per 100,000 live births in 2012. However, in the ensuing decade, congenital syphilis has resurged, and as of 2022, rates reached 57.2 per 100,000 live births [22]. Worldwide, it is reported that 7.1 million adults were newly infected with syphilis in 2020, and congenital syphilis is the second leading cause of preventable stillbirth globally [23]. As a result, pregnancy-associated syphilis remains a significant global public health concern. No vaccine is available.

### 3.2. Pathogenesis

The pathogenesis of syphilis is variable but includes three distinct stages: primary, secondary, and tertiary [3]. Primary syphilis begins with the inoculation of the skin or mucous membranes with *T. pallidum*. The bacteria replicate at the site of infection and a painless, indurated lesion called a chancre forms. This lesion will heal within 4 to 6 weeks. However, during primary syphilis, *T. pallidum* disseminates through the blood to multiple sites within the host including the skin and hair follicles, mucous membranes, brain, GI system, liver, and kidneys. This disseminated infection is secondary syphilis and produces a clinical syndrome of lymphadenopathy, muscle aches, weight loss, fatigue, and rashes. After about 3 months, symptoms of secondary syphilis resolve, and the infection enters a latent, asymptomatic stage. After a variable latency period, tertiary syphilis typically manifests as aortitis, or a constellation of neurologic complications known as neurosyphilis. Progression to this late stage of infection is rare in the post-antibiotic era, especially because antibiotic regimens for other common infections are capable of treating *T. pallidum* as well.

The study of human clinical data has shown that *T. pallidum* can invade and cross the placenta. Vertical transmission is associated with maternal early-stage infection and high antitreponemal antibody titer, which both correlate with high bacterial load [21,24]. Bacteria are visible in the placenta in a substantial percentage of congenital syphilis cases [25]. Common findings also include villitis and increased numbers of Hofbauer cells, the placental resident macrophages. It is believed that the bacteria then spread hematogenously to the developing fetus since necrotizing funisitis and spirochetes in the umbilical cord are both common findings [25,26]. *T. pallidum* can then spread systemically in the fetus, similarly to its infection in adults. A significant portion (~30%) of fetuses with congenital syphilis are stillborn [27]. Fetal demise is thought to be caused by placental damage leading to hypoxemia, as stillborn fetuses often show hepatosplenomegaly and are more likely to exhibit erythroblastosis compared to live-born congenital syphilis infants [26,28]. Those infants who survive to term can suffer from a variety of neurologic abnormalities, bone malformation, and hematopoietic failure [21]. There is no current vaccine to protect mothers or the developing fetus from syphilis, and diagnosis can be challenging even for the most experienced clinicians. Maternal treatment involves a single dose of intramuscular penicillin in the early stages of infection (primary, secondary, or early latent) or three weekly doses of penicillin for late latent or tertiary syphilis [29].

### 3.3. Bacterial Factors

Despite its clinical importance, the pathogenesis of congenital syphilis remains poorly understood. *T. pallidum* is a microaerophilic spirochete and obligate human pathogen that requires complex culturing conditions. It was not stably maintained in vitro until 2017, and there are limited animal models to study vertical transmission [30]. However, it is known that *T. pallidum*’s remarkable ability to disseminate throughout the body is critical for pathogenesis. The organism lacks the toxins or hemolysins typically associated with bacterial pathogens [3]. Nonetheless, it employs a variety of mechanisms that permit tissue invasion. *T. pallidum* produces multiple proteins that mediate bacterial attachment to fibronectin and laminin, components of the extracellular matrix [31,32]; this interaction with the extracellular matrix helps bacteria attach to endothelial cells and cross from blood vessels into host tissues [33]. Recently, Primus and coauthors were able to identify a *T. pallidum* surface lipoprotein Tp0954 that mediates attachment to placental cell lines, which suggests that there are virulence factors that specifically contribute to congenital syphilis [34]. With the recent advancement in techniques for *T. pallidum* culture and genetic manipulation, we expect knowledge of vertical transmission of this organism to rapidly increase in the coming years [35,36].

### 3.4. Immunity to T. pallidum

As with its pathogenesis, immunity to *T. pallidum* in the context of vertical transmission has not been amenable to study in any detail. However, the study of immunity to syphilis in other contexts does provide some insight into the immune response to *T. pallidum* likely to occur in the placenta. Unlike most Gram-negative bacteria, *T. pallidum* has a unique outer surface which does not contain LPS or other common immunostimulatory molecules [3]. As a result, it is only weakly taken up by host phagocytes and does not generate a sufficiently strong innate immune response to clear the bacterium [37,38]. Thus, clearance of the bacteria requires the assistance of adaptive immunity in the form of cytokine production and antibody-mediated opsonization. In fact, these two mechanisms appear to work synergistically to promote bacterial uptake and killing by professional phagocytes [39]. CD4+ and CD8+ T cells in syphilitic lesions produce IFN-γ and IL-1, Th1-type cytokines [40,41,42]. The presence of CD56+ NK cells also contribute to cytokine production in human lesions [42]. Rabbit models of syphilis indicate the importance of antibody production for bacterial clearance and infected humans are known to mount a strong antibody response [3,39,43]. As would be suggested by the chronic nature of syphilis, *T. pallidum* has developed multiple methods for escaping host adaptive immunity. Its surface is poorly immunogenic and it utilizes antigenic variation to prevent recognition by host receptors [41]. There is also evidence that syphilis increases the number and suppressive potency of T regulatory (Treg) cells [44,45]. Some of these Tregs were found to be specific to TpF1, a bacterial protein which also induces TGF-β and IL-10 production in monocytes [45]. Taken together, these findings suggest that *T. pallidum* potentiates a Treg response to prevent the development of more effective Th1 immunity.

## 4. *Listeria monocytogenes*

### 4.1. Epidemiology

*L. monocytogenes* is a Gram-positive rod found ubiquitously in the soil where it lives as a saprophyte. Because of its widespread distribution, it is a common contaminant of food including meat, dairy products, produce, and processed foods. The number of reported cases per year remains low, likely due to the limited susceptibility of healthy individuals to infection; however, some countries (such as those in the European Union or EU) have reported increases in listeriosis cases in recent years [46]. *L. monocytogenes* has as extremely high lethality rate (20–50%), making it the third leading cause of death from foodborne infection in the US [47,48]. Pregnant women are 10 to 20 times more likely to be infected with listeriosis compared to otherwise healthy adults [48]. The reason for this disparity is not known but may be related to changes in cell-mediated immunity during pregnancy [49].

The exact incidence of listeriosis in pregnancy is difficult to estimate because of variations between populations and challenges in diagnosis. Women can have nonspecific symptoms or experience pregnancy loss as the only symptom, and microbiologic testing is not routinely performed on early-term miscarriages [50]. A cohort study over 10 years in Israel estimated the incidence of pregnancy-associated listeriosis at 5–25 cases per 100,000 live births [51]. A study of outcomes for pregnancy-associated listeriosis found 83% of cases involved a major adverse pregnancy outcome and 24% resulted in fetal loss [52]. The reported incidence of neonatal listeriosis based on several studies is between 1.3 and 25 per 100,000 live births [53].

### 4.2. Pathogenesis

A classic *L. monocytogenes* infection can be divided into four general stages: replication in the intestine and invasion of intestinal cells, dissemination through the blood to the spleen and liver, replication in these target organs, and dissemination to additional organs such as the placenta. Depending on the dose and host condition, infection can either be resolved at any of the four stages or progress to uncontrolled bacteremia and sepsis [54]. Initially, the host is colonized when *L. monocytogenes* enters the gastrointestinal (GI) tract. Bacteria can invade and infiltrate intestinal epithelial cells by cellular uptake, vacuolar escape, and cytosolic replication. They can then utilize actin to spread directly between neighboring cells without contacting the extracellular milieu [4]. Eventually, *L. monocytogenes* escapes from epithelial cells and can penetrate the GI tract submucosa and enter the blood [54]. Phagocytes in the liver and spleen rapidly remove bacteria from the blood but enable the establishment of infection in these organs [55]. Subcapsular dendritic cells and marginal zone macrophages are the primary sites of splenic colonization and enable *L. monocytogenes* to reach high numbers within the spleen [55,56]. Further dissemination from the liver and spleen can then occur, most commonly to the brain and placenta. There is recent evidence that *L. monocytogenes* can also traffic from the spleen to the gallbladder, permitting fecal shedding of bacteria and potential recolonization of the GI tract.

There is no detailed information regarding how the vertical transmission of *L. monocytogenes* occurs in humans, but multiple animal models have been used to understand its pathogenesis. Because *L. monocytogenes* exhibits fetal tropism in multiple species [57], it seems likely that basic principles derived from animal models will likely be similarly applicable to human disease, at least to some degree. A study in pregnant non-human primates calculated a maternal dose of 10^7^ CFU was lethal to 50% of third-trimester fetuses. Pregnancy loss was observed at doses as low at 10^3^ CFU [58]. These bacterial numbers are consistent with exposure through food contamination. In fact, the EU permits up to 10^2^ CFU per gram of *L. monocytogenes* to be present in food at its expiration date [59]. Another primate study found that 10^7^ CFU led to fetal demise during the first trimester, indicating that *L. monocytogenes* is a danger to fetal health during the entirety of pregnancy [60]. Interestingly, inoculation did not produce any clinical signs in the mothers apart from pregnancy loss. Use of a lower dose of 10^6^ CFU at mid-gestation in primates resulted in sterile inflammation in the placenta and an increased rate of pregnancy complications without the actual transmission of bacteria to the placenta or fetus [61]. This study also found that prior maternal infection with *L. monocytogenes* was protective against these outcomes. Together, this evidence indicates that *L. monocytogenes* can produce variable clinical syndromes depending on the dose, timing of exposure, and immunologic history of the host.

Direct spread to the placenta is thought to occur via the blood, and placental infection in nonhuman primates is associated with bacteremia [60]. However, it appears that the cell types in contact with maternal blood are hostile to *L. monocytogenes* invasion. Studies using tagged bacterial strains in pregnant guinea pigs suggested that the founding population in the placenta is very small, possibly as little as one bacterium [62]. Microscopy using a pregnant mouse model indicated that bacterial invasion of the placenta first occurs in the cytotrophoblasts, which line the central arterial canal of the placenta [63]. The bacteria then replicate within this layer, generating a focus of infection that spreads outward to the rest of the placenta, eventually reaching the labyrinth and breaching the maternal-fetal barrier. These results are supported by in vitro findings using both human placental organ culture and murine trophoblast stem cells that indicate that syncytiotrophoblasts are highly resistant to invasion by *L. monocytogenes* and that infection must first become established in extravillous cytotrophoblasts [64,65,66]. One common finding in all animal models is that once infection is established, bacteria can rapidly grow to high numbers in the placenta, enabling subsequent colonization of the fetus [60,63,67]. One study in guinea pigs even found evidence that the placenta could serve as a reservoir for reseeding the spleen with *L. monocytogenes* [62].

There are limitations associated with animal models of *L. monocytogenes* infection. The architecture of the placenta varies with animal species. Experimental evidence suggests that some of the surface proteins used by *L. monocytogenes* to invade different cell types are species specific. For example, *L. monocytogenes* InlA, which binds to E-cadherin, interacts with human and guinea pig E-cadherin, but not mouse or rat [68]. InlB, which binds to the Met receptor as well as gC1qR and proteoglycans, interacts with human and mouse Met but not with guinea pig [69,70,71]. InlA and InlB both recognize gerbil receptors [72]; however, the historical lack of genetic models in gerbils means this animal model lacks the power of mouse models. Despite these limitations, the ability of *L. monocytogenes* to infect a wide range of animal species and exhibit vertical transmission suggests that at least some facets and/or pathways of infection are likely conserved across species.

### 4.3. Bacterial Factors

While recognizing some of the caveats of animal studies with regard to *L. monocytogenes* vertical transmission, research using model organisms has elucidated some of the bacterial factors that are necessary for invasion of the placenta. Multiple members of the internalin family of bacterial surface proteins have been implicated in vertical transmission. Using the gerbil model of vertical transmission mentioned above, findings indicate that coordinated action of internalin A (InlA) and internalin B (InlB) is necessary for placental invasion [72]. The data in the mouse model further indicate that, under some conditions, InlB alone may be sufficient to initiate vertical transmission [73]. Since trophoblasts exhibit high expression of both E-cadherin and c-Met, it is logical that these proteins would enhance invasion of this cell type. And because the available data implicate placental infection as being a bottleneck event, marginal increases in host cell invasion may lead to large increases in observed vertical transmission. A secreted internalin, internalin P, has also been identified as promoting invasion of the placenta [74]. Internalin P interacts with the host protein afadin to disrupt cell–cell junctions and promote transcytosis of bacteria [75].

Epidemiological evidence has long indicated that certain groups of *L. monocytogenes* strains are associated with pregnancy infections. Specifically, strains grouped into serotype IVb are overrepresented in pregnancy-associated infections [52,76,77]. An analysis of *L. monocytogenes* clinical isolates from France used whole-genome sequencing to categorize strain groups more accurately [78]. Maury and coauthors identified several clades within the IVb serotype associated with vertical transmission, including clonal complexes 4 and 6. They also identified a PTS sugar transport system that may enhance pregnancy infection. Another study demonstrated that increased expression of InlB in clonal complex 4 strains is responsible for increased vertical transmission in a mouse model [73]. Despite the strong evidence that some strains of *L. monocytogenes* are hypertransmissible, the molecular mechanisms which underlie these differences are only beginning to be elucidated.

### 4.4. Immunity to L. monocytogenes

The ability to readily culture and genetically manipulate *L. monocytogenes* as well as the availability of immune competent animal infection models means that more is known regarding the host response to *L. monocytogenes* pregnancy infections than for *T. pallidum*, and perhaps for any other vertically transmitted microorganism. A brief illustrative synopsis of what is currently known for *L. monocytogenes* pregnancy infections resulting from animal infection models follows. In non-pregnant hosts, the adaptive immune response is necessary for the clearance of *L. monocytogenes* infection. Both CD4+ and CD8+ T cells are part of the antilisterial response. However, because *L. monocytogenes* is an intracellular pathogen, CD8+ T cells appear to be the more important subset [79]. The critical CD8+ T cell effector functions appear to be direct killing of infected cells and the production of IFN-γ [80].

A study in several mouse strains found no difference in CD8+ T cell responses between pregnant and non-pregnant animals. In both groups, the responses included anti-*Listeria* CD8+ T cells that produce IFN-γ. In fact, the pregnant animals exhibited more rapid clearance of the infection [81]. However, there is evidence that pregnancy can alter the systemic immune response to *L. monocytogenes*. Pregnancy reduces the protection provided by prior *L. monocytogenes* infection. Despite this loss of protection, there was no change in the number or function of anti-*L. monocytogenes* CD8+ T cells [82]. Interestingly, when mice were mated with syngeneic mice, i.e., mice from the same inbred strain, there was no defect in immune protection during pregnancy [82]. A recently published study found that pregnancy also altered the glycosylation of anti-*L. monocytogenes* antibodies so as to better protect neonatal mice against *L. monocytogenes* infection [83].

Bacterial entry into the placenta also activates innate defense mechanisms within the placenta. A recent study identified decidual NK (dNK) cells as part of the immunity against *L. monocytogenes* infection [84]. The cells transfer the antimicrobial peptide granulysin to infected trophoblasts without inducing the standard granzyme-B-mediated cytotoxicity. This is because the dNKs did not degranulate or form an immune synapse but instead transferred the granulysin via cytoplasmic bridges. Mice do not produce granulysin, which limits the study of this phenomenon in many vivo models of vertical transmission. However, expression of granulysin transgenically reduced the rate of pregnancy loss in infected mice.

In vitro evidence indicates that infection with *L. monocytogenes* causes trophoblasts to produce proinflammatory cytokines such as TNF-α and IL-6 in addition to chemokines CXCL8, CCL3, and CCL4 [85]. Extracellular vesicles from infected trophoblasts also communicate the presence of bacteria and can induce TNF-α production in naïve cells [86]. Interestingly, trophoblasts continue to produce IL-27, IL-10, and IL-1RA, immunosuppressive cytokines important for fetal tolerance, even after *L. monocytogenes* infection [85]. Hofbauer cells, placental resident macrophages, can be infected by and potentially even spread *L. monocytogenes* [87]. They respond to infection by transitioning from an M2 to M1 phenotype, producing high levels of inflammatory cytokines including inflammasome dependent IL-1β and IL-18. They also produce numerous chemokines including CXCL8, CXCL10, CCL3, and CCL4. Similar to trophoblasts cells, Hofbauer cells maintain or increase the production of signaling molecules associated with T cell tolerance even during infection with *L. monocytogenes*. Further, they are not observed to produce CD80, an important costimulatory molecule for antigen presentation to T cells.

The observation that the trophoblasts and Hofbauer cells continue to produce protolerance cytokines during *L. monocytogenes* infection emphasizes the need to maintain fetal tolerance even during microbial infection. Pregnant mice with allogeneic fetuses have increased numbers of immunosuppressive FoxP3+ Treg cells [88]. These increased Tregs cause increased bacterial burdens in the maternal liver and spleen. The increased infection susceptibility appears to be driven by Treg production of IL-10, which is required for maintenance of pregnancy. However, tolerance can be disrupted by *L. monocytogenes* infection. The infection of pregnant mice produces dose-dependent fetal loss [89]. While some resorbed fetoplacental units are directly infected with *L. monocytogenes*, others are lost due to autoimmune inflammation. During infection, neutrophils and macrophages are recruited to the placenta via placental chemokine production [90]. These cells then secrete CXCL9, which recruits fetal specific CD8+ T cells. This anti-fetal T cell response leads to severe inflammation and fetal loss. As part of this process, *L. monocytogenes* infection reduces the ability of maternal Tregs to suppress T cell responses and causes upregulation of CXCR3, the receptor for CXCL9, on CD8+ Tregs [88,90].

**Figure 1:**
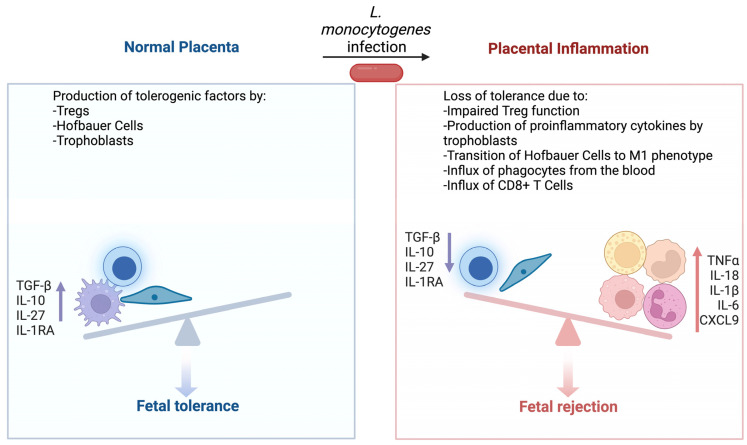
Placental inflammation leads to loss of fetal tolerance. During normal development, placental Hofbauer cells, trophoblasts, and Tregs prevent the development of anti-fetal immunity. These cells maintain the production of tolerogenic factors even during infection with *L. monocytogenes*. However, a sufficiently severe infection eventually leads to increased proinflammatory cytokines, transition of Hofbauer cells towards an M1 phenotype, and an influx of innate immune cells. These processes culminate in loss of Treg function and the development of anti-fetal CD8+ T cells. Figure created using BioRender.

Thus, the study of placental immunity to *L. monocytogenes* provides insight into the central tension of fetal tolerance vs. pathogen resistance (Figure 1). Native placental cell types produce proinflammatory cytokines in response to bacterial infections and continue to produce tolerogenic factors which normally prevent T cell activation. However, *L. monocytogenes* can avoid innate immunity and thus T cell immunity is necessary for clearance. As a result, the bacteria can rapidly grow to high numbers within an infected placenta. This infection then leads to an influx of neutrophils and monocytes from the blood, which promote T cell activation. However, the generation of anti-*Listeria* T cells also produces anti-fetal CD8+ T cells. Once fetal tolerance has been fractured, the cycle of inflammation escalates and eventually results in pregnancy loss.

## 5. Preventative Measures against Fetal Infection for *T. pallidum* and *L. monocytogenes* and a Comparison of Managing Infections

Currently, there are no vaccines for the prevention of either *T. pallidum* or *L. monocytogenes* infection, and antimicrobial treatment remains the best option for treating active infections. For *T. pallidum* infections, the past decade has seen increased rates of congenital syphilis with the highest incidence in low- and middle-income countries [91]. Eliminating the vertical transmission of syphilis requires a global commitment, and a reduction in congenital infections requires repeat testing in high-risk pregnancies, partner screening, point-of-care testing, and a concerted effort in tackling health inequalities affecting marginalized populations [92]. The standard treatment for congenital syphilis is 10 days of intravenous benzylpenicillin (every 12 h during the first 7 days of life, and every 8 h thereafter for a total of 10 days) [92]. Untreated syphilis during pregnancy, especially during the early stages of infection, has an estimated 60% risk of negative birth outcomes [93]. For *L. monocytogenes* infections during pregnancy, detection is challenging as often the mother has unremarkable febrile symptoms or even no symptoms, and early diagnosis is difficult [94]. Pregnant women suspected of harboring a *L. monocytogenes* infection should be treated with antimicrobials, with intravenous amoxicillin or ampicillin being the drugs of first choice given their ability to penetrate the placental barrier [95]. While infections caused by *L. monocytogenes* occur via contaminated food products, it is difficult if not impossible to completely prevent infection; however, high-risk foods such as deli meats, unpasteurized dairy products, and foods with extended shelf lives should be avoided [96]. Overall, a greater understanding of how *T. pallidum* and *L. monocytogenes* penetrate the placental barrier and establish infection is needed to provide additional strategies for treating and preventing such infections.

## 6. Conclusions

**Figure 2:**
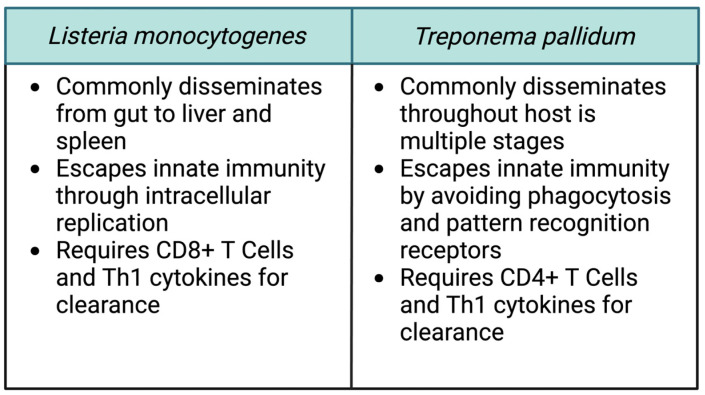
Common features of vertically transmitted pathogens. Despite the many apparent differences between *Listeria monocytogenes* and *Treponema pallidum*, a careful comparison of the organisms reveals some common features which contribute to their ability to invade and cross the placenta. Figure created using BioRender.

Comparing the pathogenesis of these two bacteria can provide some insight into the process of transplacental transmission (Figure 2). Even for these vertically transmitted pathogens, the placental tissue itself forms a formidable barrier to infection. For both pathogens, not all infections during pregnancy result in transmission, and experimental evidence indicates that *L. monocytogenes* infection in the placenta begins with an extremely small number of organisms. This fact suggests why so few bacterial species cause pregnancy-associated infections. Both *L. monocytogenes* and *T. pallidum* commonly spread to secondary target organs within the host as a part of their life cycle. As a result, they have each evolved multiple mechanisms that enable them to not just cause infection, but to disseminate within the host and actively invade uncolonized tissues. Only with the aid of those mechanisms can the bacteria cross the placental barrier and take advantage of the fetal niche. This lifestyle stands in contrast to other common bacterial pathogens. For example, *Staphylococcus aureus* commonly causes localized tissue infection. While it can disseminate, it typically does so in the late stages of a severe infection.

It is also notable that host control of both *T. pallidum* and *L. monocytogenes* requires the functioning of adaptive immunity. The role of CD8+ T cells in the clearance of *L. monocytogenes* has been well defined, and *T. pallidum* typically causes a chronic infection resistant to immune clearance. The experimental data indicate that the placental immune response suppresses the development of T cell immunity to prevent the development of fetal rejection. Thus, bacteria capable of evading innate immunity and for which bacterial dissemination is a key facet of disease, such as for *T. pallidum* and *L. monocytogenes*, will be able to persist in the placenta and cause fetal infection. T cell activation sufficient to kill the pathogen appears to inevitably lead to anti-fetal T cells, spiraling inflammation, and pregnancy loss. This observation also provides a potential clue as to why many more viruses can be vertically transmitted than bacteria. Since adaptive immunity, and CD8+ T cells in particular, are critical for clearing viruses, they would be capable of taking advantage of the placental niche which actively suppresses such responses.

Vertically transmitted infections remain a serious public health problem, and our knowledge of their pathogenesis remains limited. However, new advances in our understanding of transplacentally transmitted bacteria promise to help elucidate how complex tissue architecture, a unique immune environment, and microbial virulence combine to produce adverse clinical outcomes.

## References

[1] Cunningham F.G., Leveno K.J., Dashe J.S., Hoffman B.L., Spong C.Y., Casey B.M. (2022). Infectious Diseases. Williams Obstetrics.

[2] Cunningham F.G., Leveno K.J., Dashe J.S., Hoffman B.L., Spong C.Y., Casey B.M. (2022). Sexually Transmitted Infections. Williams Obstetrics.

[3] Lafond R.E., Lukehart S.A. (2006). Biological Basis for Syphilis. Clin. Microbiol. Rev..

[4] Freitag N.E., Port G.C., Miner M.D. (2009). *Listeria monocytogenes*—From saprophyte to intracellular pathogen. Nat. Rev. Microbiol..

[5] Erlebacher A. (2013). Immunology of the Maternal-Fetal Interface. Annu. Rev. Immunol..

[6] Crespo Â.C., van der Zwan A., Ramalho-Santos J., Strominger J.L., Tilburgs T. (2016). Cytotoxic potential of decidual NK cells and CD8+ T cells awakened by infections. J. Reprod. Immunol..

[7] Koopman L.A., Kopcow H.D., Rybalov B., Boyson J.E., Orange J.S., Schatz F., Masch R., Lockwood C.J., Schachter A.D., Park P.J. (2003). Human Decidual Natural Killer Cells Are a Unique NK Cell Subset with Immunomodulatory Potential. J. Exp. Med..

[8] Kopcow H.D., Allan D.S.J., Chen X., Rybalov B., Andzelm M.M., Ge B., Strominger J.L. (2005). Human Decidual NK Cells Form Immature Activating Synapses and Are Not Cytotoxic. Proc. Natl. Acad. Sci. USA.

[9] Tilburgs T., Evans J.H., Crespo Â.C., Strominger J.L. (2015). The HLA-G cycle provides for both NK tolerance and immunity at the maternal–fetal interface. Proc. Natl. Acad. Sci. USA.

[10] Vujaklija D.V., Gulic T., Sucic S., Nagata K., Ogawa K., Laskarin G., Saito S., Haller H., Rukavina D. (2011). First Trimester Pregnancy Decidual Natural Killer Cells Contain and Spontaneously Release High Quantities of Granulysin. Am. J. Reprod. Immunol..

[11] Liu L., Huang X., Xu C., Chen C., Zhao W., Li D., Li L., Wang L., Du M. (2020). Decidual CD8+T cells exhibit both residency and tolerance signatures modulated by decidual stromal cells. J. Transl. Med..

[12] Van Der Zwan A., Bi K., Norwitz E.R., Crespo Â.C., Claas F.H.J., Strominger J.L., Tilburgs T. (2017). Mixed signature of activation and dysfunction allows human decidual CD8 + T cells to provide both tolerance and immunity. Proc. Natl. Acad. Sci. USA.

[13] Scaife P.J., Bulmer J.N., Robson S.C., Innes B.A., Searle R.F. (2006). Effector Activity of Decidual CD8+ T Lymphocytes in Early Human Pregnancy. Biol. Reprod..

[14] Ingman K., Cookson V.J.K.W., Jones C.J.P., Aplin J.D. (2010). Characterisation of Hofbauer Cells in First and Second Trimester Placenta: Incidence, Phenotype, Survival in vitro and Motility. Placenta.

[15] Kim J., Romero R., Kim M.R., Kim Y.M., Friel L., Espinoza J., Kim C.J. (2008). Involvement of Hofbauer cells and maternal T cells in villitis of unknown aetiology. Histopathology.

[16] Castellucci M., Zaccheo D., Pescetto G. (1980). A three-dimensional study of the normal human placental villous core. I. The Hofbauer cells. Cell Tissue Res..

[17] Anteby E.Y., Natanson-Yaron S., Greenfield C., Goldman-Wohl D., Haimov-Kochman R., Holzer H., Yagel S. (2005). Human Placental Hofbauer Cells Express Sprouty Proteins: A Possible Modulating Mechanism of Villous Branching. Placenta.

[18] Demir R., Kayisli U.A., Seval Y., Celik-Ozenci C., Korgun E.T., Demir-Weusten A.Y., Huppertz B. (2004). Sequential Expression of VEGF and its Receptors in Human Placental Villi During Very Early Pregnancy: Differences Between Placental Vasculogenesis and Angiogenesis. Placenta.

[19] Ahmed A., Li X.F., Dunk C., Whittle M.J., Rushton D.I., Rollason T. (1995). Colocalisation of Vascular Endothelial Growth Factor and Its Flt-1 Receptor in Human Placenta. Growth Factors.

[20] Reyes L., Golos T.G. (2018). Hofbauer Cells: Their Role in Healthy and Complicated Pregnancy. Front. Immunol..

[21] Adhikari E.H. (2020). Syphilis in Pregnancy. Obstet. Gynecol..

[22] (2022). Division of STD Prevention Sexually Transmitted Disease Surveillance, 2021.

[23] (2022). Global Report on HIV, Viral Hepatitis, and Sexually Transmitted Infections, 2021.

[24] Wang Y., Wu M., Gong X., Zhao L., Zhao J., Zhu C., Gong C. (2019). Risk Factors for Congenital Syphilis Transmitted from Mother to Infant—Suzhou, China, 2011–2014. MMWR Morb. Mortal. Wkly. Rep..

[25] Kittipornpechdee N., Hanamornroongruang S., Lekmak D., Treetipsatit J. (2018). Fetal and Placental Pathology in Congenital Syphilis: A Comprehensive Study in Perinatal Autopsy. Fetal Pediatr. Pathol..

[26] Sheffield J.S., Sánchez P.J., Wendel G.D., Fong D.W.I., Margraf L.R., Zeray F., Mcintire D.D., Rogers B.B. (2002). Placental histopathology of congenital syphilis. Obstet. Gynecol..

[27] Garel B., Grange P., Benhaddou N., Schaub B., Desbois-Nogard N., Thouvenin M., Lepoutre X., Levy R., Navarro C., Charlier C. (2019). Congenital syphilis: A prospective study of 22 cases diagnosed by PCR. Ann. Dermatol. Vénéréol..

[28] Young S.A., Crocker D.W. (1994). Occult congenital syphilis in macerated stillborn fetuses. Arch. Pathol. Lab. Med..

[29] Sankaran D., Partridge E., Lakshminrusimha S. (2023). Congenital Syplilis an Illustrative Review. Children.

[30] Edmondson D.G., Hu B., Norris S.J. (2018). Long-Term In Vitro Culture of the Syphilis Spirochete *Treponema pallidum* subsp. *pallidum*. mBio.

[31] Cameron C.E., Brown E.L., Kuroiwa J.M.Y., Schnapp L.M., Brouwer N.L. (2004). *Treponema pallidum* Fibronectin-Binding Proteins. J. Bacteriol..

[32] Cameron C.E. (2003). Identification of a *Treponema pallidum* Laminin-Binding Protein. Infect. Immun..

[33] Lee J.H., Choi H.J., Jung J., Lee M.G., Lee J.B., Lee K.H. (2003). Receptors for *Treponema pallidum* Attachment to the Surface and Matrix Proteins of Cultured Human Dermal Microvascular Endothelial Cells. Yonsei Med. J..

[34] Primus S., Rocha S.C., Giacani L., Parveen N. (2020). Identification and Functional Assessment of the First Placental Adhesin of *Treponema pallidum* That May Play Critical Role in Congenital Syphilis. Front. Microbiol..

[35] Edmondson D.G., Norris S.J. (2021). In Vitro Cultivation of the Syphilis Spirochete *Treponema pallidum*. Curr. Protoc..

[36] Phan A., Romeis E., Tantalo L., Giacani L. (2022). In Vitro Transformation and Selection of *Treponema pallidum* subsp. *pallidum*. Curr. Protoc..

[37] Moore M.W., Cruz A.R., LaVake C.J., Marzo A.L., Eggers C.H., Salazar J.C., Radolf J.D. (2007). Phagocytosis of *Borrelia burgdorferi* and *Treponema pallidum* Potentiates Innate Immune Activation and Induces Gamma Interferon Production. Infect. Immun..

[38] Sellati T.J., Bouis D.A., Kitchens R.L., Darveau R.P., Pugin J., Ulevitch R.J., Gangloff S.C., Goyert S.M., Norgard M.V., Radolf J.D. (1998). *Treponema pallidum* and *Borrelia burgdorferi* Lipoproteins and Synthetic Lipopeptides Activate Monocytic Cells via a CD14-Dependent Pathway Distinct from That Used by Lipopolysaccharide. J. Immunol..

[39] Hawley K.L., Cruz A.R., Benjamin S.J., La Vake C.J., Cervantes J.L., Ledoyt M., Ramirez L.G., Mandich D., Fiel-Gan M., Caimano M.J. (2017). IFNγ Enhances CD64-Potentiated Phagocytosis of *Treponema pallidum* Opsonized with Human Syphilitic Serum by Human Macrophages. Front. Immunol..

[40] van Voorhis W.C., Barrett L.K., Koelle D.M., Nasio J.M., Plummer F.A., Lukehart S.A. (1996). Primary and Secondary Syphilis Lesions Contain mRNA for Th1 Cytokines. J. Infect. Dis..

[41] Stary G.G., Klein I.I., Brüggen M.M., Kohlhofer S.S., Brunner P.M.P.M., Spazierer D.D., Müllauer L.L., Petzelbauer P.P., Stingl G.G. (2010). Host Defense Mechanisms in Secondary Syphilitic Lesions. Am. J. Pathol..

[42] Cruz A.R., Ramirez L.G., Zuluaga A.V., Pillay A., Abreu C., Valencia C.A., La Vake C., Cervantes J.L., Dunham-Ems S., Cartun R. (2012). Immune Evasion and Recognition of the Syphilis Spirochete in Blood and Skin of Secondary Syphilis Patients: Two Immunologically Distinct Compartments. PLoS Neglect. Trop. Dis..

[43] Salazar J.C., Hazlett K.R.O., Radolf J.D. (2002). The immune response to infection with *Treponema pallidum*, the stealth pathogen. Microbes Infect..

[44] Li K., Wang C., Lu H., Gu X., Guan Z., Zhou P. (2013). Regulatory T Cells in Peripheral Blood and Cerebrospinal Fluid of Syphilis Patients with and without Neurological Involvement. PLoS Negl. Trop. Dis..

[45] Babolin C., Amedei A., Ozolins D., Zilevica A., D’Elios M.M., de Bernard M. (2011). TpF1 from *Treponema pallidum* Activates Inflammasome and Promotes the Development of Regulatory T Cells. J. Immunol..

[46] Hadjicharalambous C., Grispoldi L., Chalias T., Cenci-Goga B. (2022). A quantitative risk assessment of *Listeria monocytogenes* from prevalence and concentration data: Application to a traditional read to eat (RTE) meat product. Int. J. Food Microbiol..

[47] Hoffmann S., Maculloch B., Batz M. (2015). Economic burden of major foodborne illnesses acquired in the United States. Economic Cost of Foodborne Illnesses in the United States.

[48] Frieden T.R., Harold Jaffe D.W., Cardo D.M., Moolenaar R.L., Leahy M.A., Martinroe J.C., Spriggs S.R., Starr T.M., Doan Q.M., King P.H. (2013). Vital Signs: Listeria Illnesses, Deaths, and Outbreaks—United States, 2009–2011. Ann. Emerg. Med..

[49] Mateus T., Silva J., Maia R.L., Teixeira P. (2013). Listeriosis during Pregnancy: A Public Health Concern. ISRN Obstet. Gynecol..

[50] Madjunkov M., Chaudhry S., Ito S. (2017). Listeriosis during pregnancy. Arch. Gynecol. Obstet..

[51] Elinav H., Hershko-Klement A., Valinsky L., Jaffe J., Wiseman A., Shimon H., Braun E., Paitan Y., Block C., Sorek R. (2014). Pregnancy-Associated Listeriosis: Clinical Characteristics and Geospatial Analysis of a 10-Year Period in Israel. Clin. Infect. Dis..

[52] Charlier C., Perrodeau É., Leclercq A., Cazenave B., Pilmis B., Henry B., Lopes A., Maury M.M., Moura A., Goffinet F. (2017). Clinical features and prognostic factors of listeriosis: The MONALISA national prospective cohort study. Lancet Infect. Dis..

[53] Koopmans Merel M., Brouwer Matthijs C., Vázquez-Boland José A., van de Beek D. (2022). Human Listeriosis. Clin. Microbiol. Rev..

[54] Regan T., MacSharry J., Brint E. (2014). Tracing innate immune defences along the path of *Listeria monocytogenes* infection. Immunol. Cell Biol..

[55] Waite J.C., Leiner I., Lauer P., Rae C.S., Barbet G., Zheng H., Portnoy D.A., Pamer E.G., Dustin M.L. (2011). Dynamic imaging of the effector immune response to listeria infection In Vivo. PLoS Pathog..

[56] Aoshi T., Carrero J.A., Konjufca V., Koide Y., Unanue E.R., Miller M.J. (2009). The cellular niche of *Listeria monocytogenes* infection changes rapidly in the spleen. Eur. J. Immunol..

[57] Lamond N., Freitag N. (2018). Vertical Transmission of *Listeria monocytogenes*: Probing the Balance between Protection from Pathogens and Fetal Tolerance. Pathogens.

[58] Smith M.A., Takeuchi K., Anderson G., Ware G.O., McClure H.M., Raybourne R.B., Mytle N., Doyle M.P. (2008). Dose-Response Model for *Listeria monocytogenes*-Induced Stillbirths in Nonhuman Primates. Infect. Immun..

[59] The Commission of the European Communities. https://eur-lex.europa.eu/legal-content/EN/TXT/PDF/?uri=CELEX:02005R2073-20140601&rid=1.

[60] Wolfe B., Wiepz G.J., Schotzko M., Bondarenko G.I., Durning M., Simmons H.A., Mejia A., Faith N.G., Sampene E., Suresh M. (2017). Acute Fetal Demise with First Trimester Maternal Infection Resulting from *Listeria monocytogenes* in a Nonhuman Primate Model. mBio.

[61] Wolfe B., Kerr A.R., Mejia A., Simmons H.A., Czuprynski C.J., Golos T.G. (2019). Sequelae of Fetal Infection in a Non-human Primate Model of Listeriosis. Front. Microbiol..

[62] Bakardjiev A.I., Theriot J.A., Portnoy D.A. (2006). *Listeria monocytogenes* Traffics from Maternal Organs to the Placenta and Back. PLoS Pathog..

[63] Le Monnier A., Join-Lambert O.F., Jaubert F., Berche P., Kayal S. (2006). Invasion of the Placenta during Murine Listeriosis. Infect. Immun..

[64] Robbins J.R., Skrzypczynska K.M., Zeldovich V.B., Kapidzic M., Bakardjiev A.I. (2010). Placental syncytiotrophoblast constitutes a major barrier to vertical transmission of *Listeria monocytogenes*. PLoS Pathog..

[65] Zeldovich V.B., Clausen C.H., Bradford E., Fletcher D.A., Maltepe E., Robbins J.R., Bakardjiev A.I. (2013). Placental Syncytium Forms a Biophysical Barrier against Pathogen Invasion. PLoS Pathog..

[66] Zeldovich V.B., Robbins J.R., Kapidzic M., Lauer P., Bakardjiev A.I. (2011). Invasive Extravillous Trophoblasts Restrict Intracellular Growth and Spread of *Listeria monocytogenes*. PLoS Pathog..

[67] Bakardjiev A.I., Stacy B.A., Portnoy D.A. (2005). Growth of *Listeria monocytogenes* in the Guinea Pig Placenta and Role of Cell-to-Cell Spread in Fetal Infection. J. Infect. Dis..

[68] Mengaud J., Ohayon H., Gounon P., Mège R., Cossart P. (1996). E-Cadherin Is the Receptor for Internalin, a Surface Protein Required for Entry of *L. monocytogenes* into Epithelial Cells. Cell.

[69] Shen Y., Naujokas M., Park M., Ireton K. (2000). InlB-Dependent Internalization of Listeria Is Mediated by the Met Receptor Tyrosine Kinase. Cell.

[70] Braun L., Ghebrehiwet B., Cossart P. (2000). gC1q-R/p32, a C1q-binding protein, is a receptor for the InlB invasion protein of *Listeria monocytogenes*. EMBO J..

[71] Jonquières R., Pizarro-Cerdá J., Cossart P. (2001). Synergy between the N- and C-terminal domains of InlB for efficient invasion of non-phagocytic cells by *Listeria monocytogenes*. Mol. Microbiol..

[72] Disson O., Grayo S., Huillet E., Nikitas G., Langa-Vives F., Dussurget O., Ragon M., Le Monnier A., Babinet C., Cossart P. (2008). Conjugated action of two species-specific invasion proteins for fetoplacental listeriosis. Nature.

[73] Lamond N.M., McMullen P.D., Paramasvaran D., Visvahabrathy L., Eallanardo S.J., Maheswhari A., Freitag N.E. (2020). Cardiotropic isolates of *Listeria monocytogenes* with enhanced vertical transmission dependent upon the bacterial surface protein InlB. Infect. Immun..

[74] Faralla C., Rizzuto G.A., Lowe D.E., Kim B., Cooke C., Shiow L.R., Bakardjiev A.I. (2016). InlP, a New Virulence Factor with Strong Placental Tropism. Infect. Immun..

[75] Faralla C., Bastounis E.E., Ortega F.E., Light S.H., Rizzuto G., Nocadello S., Anderson W.F., Robbins J.R., Theriot J.A., Bakardjiev A.I. (2018). *Listeria monocytogenes* InlP interacts with afadin and facilitates basement membrane crossing. PLoS Pathog..

[76] McLauchlin J. (1990). Distribution of serovars of *Listeria monocytogenes* isolated from different categories of patients with listeriosis. Eur. J. Clin. Microbiol. Infect. Dis..

[77] Vasilev V., Japheth R., Andorn N., Yshai R., Agmon V., Gazit E., Kashi Y., Cohen D. (2009). A survey of laboratory-confirmed isolates of invasive listeriosis in Israel, 1997–2007. Epidemiol. Infect..

[78] Maury M.M., Tsai Y.H., Charlier C., Touchon M., Chenal-Francisque V., Leclercq A., Criscuolo A., Gaultier C., Roussel S., Brisabois A. (2016). Uncovering *Listeria monocytogenes* hypervirulence by harnessing its biodiversity. Nat. Genet..

[79] Zenewicz L.A., Shen H. (2007). Innate and adaptive immune responses to *Listeria monocytogenes*: A short overview. Microb. Infect..

[80] Condotta S.A., Richer M.J., Badovinac V.P., Harty J.T. (2012). Probing CD8 T cell responses with *Listeria monocytogenes* infection. Adv. Immunol..

[81] Krishnan L., Pejcic-Karapetrovic B., Gurnani K., Zafer A., Sad S. (2010). Pregnancy Does not Deter the Development of a Potent Maternal Protective CD8+ T-Cell Acquired Immune Response Against *Listeria monocytogenes* Despite Preferential Placental Colonization. Am. J. Reprod. Immunol..

[82] Clark D.R., Chaturvedi V., Kinder J.M., Jiang T.T., Xin L., Ertelt J.M., Way S.S. (2014). Perinatal *Listeria monocytogenes* susceptibility despite preconceptual priming and maintenance of pathogen-specific CD8+ T cells during pregnancy. Cell. Mol. Immunol..

[83] Erickson J.J., Archer-Hartmann S., Yarawsky A.E., Miller J.L.C., Seveau S., Shao T., Severance A.L., Miller-Handley H., Wu Y., Pham G. (2022). Pregnancy enables antibody protection against intracellular infection. Nature.

[84] Crespo Â.C., Mulik S., Dotiwala F., Ansara J.A., Sen Santara S., Ingersoll K., Ovies C., Junqueira C., Tilburgs T., Strominger J.L. (2020). Decidual NK Cells Transfer Granulysin to Selectively Kill Bacteria in Trophoblasts. Cell.

[85] Johnson L.J., Azari S., Webb A., Zhang X., Gavrilin M.A., Marshall J.M., Rood K., Seveau S. (2021). Human Placental Trophoblasts Infected by *Listeria monocytogenes* Undergo a Pro-Inflammatory Switch Associated With Poor Pregnancy Outcomes. Front. Immunol..

[86] Kaletka J., Lee K.H., Altman J., Kanada M., Hardy J.W. (2022). *Listeria monocytogenes* Infection Alters the Content and Function of Extracellular Vesicles Produced by Trophoblast Stem Cells. Infect. Immun..

[87] Azari S., Johnson L.J., Webb A., Kozlowski S.M., Zhang X., Rood K., Amer A., Seveau S. (2021). Hofbauer Cells Spread *Listeria monocytogenes* among Placental Cells and Undergo Pro-Inflammatory Reprogramming while Retaining Production of Tolerogenic Factors. mBio.

[88] Rowe J.H., Ertelt J.M., Aguilera M.N., Farrar M.A., Way S.S. (2011). Foxp3+ Regulatory T Cell Expansion Required for Sustaining Pregnancy Compromises Host Defense against Prenatal Bacterial Pathogens. Cell Host Microbe.

[89] Rowe J.H., Ertelt J.M., Xin L., Way S.S. (2012). *Listeria monocytogenes* Cytoplasmic Entry Induces Fetal Wastage by Disrupting Maternal Foxp3+ Regulatory T Cell-Sustained Fetal Tolerance. PLoS Pathog..

[90] Chaturvedi V., Ertelt J.M., Jiang T.T., Kinder J.M., Xin L., Owens K.J., Jones H.N., Way S.S. (2015). CXCR3 blockade protects against *Listeria monocytogenes* infection–induced fetal wastage. J. Clin. Investig..

[91] (2022). Congenital Syphilis: Sexually Transmitted Infection Treatment Guidelines.

[92] Moseley P., Bamford A., Eisen S., Lyall H., Kingston M., Thorne C., Piñera C., Rabie H., Prendergast A.J., Kadambari S. (2023). Resurgence of congenital syphilis: New strategies against an old foe. Lancet Infect. Dis..

[93] Gomez G.B., Kamb M.L., Newman L.M., Mark J., Broutet N., Hawkes S.J. (2013). Untreated maternal syphilis and adverse outcomes of pregnancy: A systematic review and meta-analysis. Bull. World Health Organ..

[94] Mylonakis E., Paliou M., Hohmann E.L., Calderwood S.B., Wing E.J. (2002). Listeriosis during pregnancy: A case series and review of 222 cases. Medicine.

[95] Kaistone C. (1991). Successful antepartum treatment of listeriosis. Am. J. Obstet. Gynecol..

[96] Allerberger F., Huhulescu S. (2015). Pregnancy related listeriosis: Treatment and control. Expert Rev. Anti-Infect. Ther..

